# High-Risk Human Papillomavirus and Other Sexually Transmitted Infections Among Women Living With Human Immunodeficiency Virus in Jos, Nigeria

**DOI:** 10.7759/cureus.103298

**Published:** 2026-02-09

**Authors:** Nanma T Cosmas, Francis A Magaji, Shedrach Y Acheng, Lipigwe Lauya, Mark O Okolo, Elizabeth N Christian, Robert L Murphy, Lifang Hou, Chad J Achenbach, Jonah Musa

**Affiliations:** 1 Department of Medical Microbiology, College of Health Sciences, University of Jos, Jos, NGA; 2 Department of Obstetrics and Gynecology, University of Jos/Jos University Teaching Hospital, Jos, NGA; 3 Genomics and Postgraduate Research Laboratory, College of Health Sciences, University of Jos, Jos, NGA; 4 Division of Human Virology, Miradi Ltd, Jos, NGA; 5 Department of Medical Microbiology, University of Jos/Jos University Teaching Hospital, Jos, NGA; 6 Robert J. Havey MD Institute for Global Health, Northwestern University Feinberg School of Medicine, Chicago, USA; 7 Division of Infectious Diseases, Department of Medicine, Northwestern University Feinberg School of Medicine, Chicago, USA; 8 Center for Global Oncology, Institute for Global Health, Northwestern University Feinberg School of Medicine, Chicago, USA; 9 Department of Preventive Medicine, Northwestern University Feinberg School of Medicine, Chicago, USA; 10 Department of Medicine, Northwestern University Feinberg School of Medicine, Chicago, USA

**Keywords:** essential stis, genital ulcers causing stis, hiv-positive women, hrhpv infection, jos, nigeria

## Abstract

Background

Women living with human immunodeficiency virus (WLWH) have a higher risk of acquiring human papillomavirus (HPV) and other sexually transmitted infections (STIs) due to their compromised immune systems. The presence of HPV, HIV, and other STIs has been implicated in cervical carcinogenesis. This study sought to understand and estimate the association between HPV infection and other STIs among HIV-positive women with and without cervical precancer.

Methodology

This study looked at women living with HIV who had been diagnosed with either low- or high-grade squamous intraepithelial lesions. This study was a part of the U54 cervical cancer project in Jos, Nigeria, from August 2019 to January 2022. Cervical swab samples were used for DNA extraction and genotyping using the HPV 28 Anyplex II (Seegene). To identify seven of the most common STIs, we used the Allplex™ STI Essential Assay (Seegene) and the Allplex™ Genital Ulcer Assay (Seegene) for seven ulcer-causing microbes.

Results

In total, 101 women were included in this study. The median age of the participants was 50 years (interquartile range = 43-57). The overall HPV prevalence was 65.3% (66/101). Among these, hrHPV infection occurred in 40.0% of the women in the age group of <40 years and 31.0% in the age group of ≥40 years. Approximately 40% of hrHPV-positive women were co-infected with at least one STI (p = 0.006). Having multiple STIs was associated with hrHPV infection (p = 0.019 for multiple essential STIs and p = 0.007 for multiple genital ulcers causing STIs). On univariate analysis, we found hrHPV to be associated at 95% confidence interval with any essential STI (p = 0.008), a single STI (p = 0.016), any genital ulcer microbe (p = 0.003), herpes simplex virus 1 (p = 0.043), *Lymphogranuloma venereum* (p = 0.017), and having a single ulcer-causing microbe (p = 0.004). In multivariate models adjusted for age, parity, HIV duration, and cervical neoplastic lesions, the odds ratio comparing hrHPV-positive to hrHPV-negative women was 10.4 (p < 0.001) for any genital ulcers causing microbial infections.

Conclusions

High-risk HPV infection was associated with the presence of other STIs in WLWH in Nigeria. Testing for STIs in women with hrHPV could have an impact on hrHPV-related carcinogenesis.

## Introduction

Human immunodeficiency virus (HIV)-positive women are at risk for infection with human papillomavirus (HPV) and other sexually transmitted infections (STIs) as they share a common primary route of transmission [1,2]. HPV is the most common viral STI in both men and women, affecting approximately 80% of the world’s sexually active population [3]. Although HPV is a cause of cervical cancer (CC), it is insufficient for the carcinogenic process [4]. Other sexually transmitted coinfections may contribute to the risk of persistent infections and cervical pre-cancer progression [5-7]. HIV infection has been shown to significantly increase a woman’s likelihood of developing CC by suppressing the ability of the immune system to clear HPV infection [8,9]. HPV and HIV synergistically favor each other in affecting the infected cells [10]. This results in HPV infection persistence and subsequent progression to precancerous lesions and cancer. Coinfection with other STIs can compound the synergistic relationship between HPV and HIV.

The association between other STIs and HPV infection is based on the cellular changes and inflammatory reactions produced by other STIs, which facilitate the proliferation and persistence of HPV infection [11]. Coinfection with *Chlamydia trachomatis*, herpes simplex virus (HSV) type 2, and HIV has been identified as an established cofactor necessary for HPV infection, persistence, and progression from cervical HPV infection to cancer [5-9]. Research has shown that STIs causing both ulcerative and non-ulcerative diseases increase the risk of HIV acquisition and contribute to cervical oncogenesis [5-7]. Additionally, the alterations caused by STIs increase the vulnerability of the cervical epithelium to genetic changes by activating oncogenes, deactivating tumor suppressor proteins, and enabling HPV to promote the development of tumor lesions [12].

There is limited data on the coinfection of HPV and other STIs among HIV-positive women in Nigeria. In this pilot study, we aimed to examine the association between hrHPV and other STIs, estimate the prevalence of HPV/STI coinfection, determine which risk factors are involved, and make recommendations for prevention, early detection, and improving health outcomes.

## Materials and methods

Study design and population

This cross-sectional study was conducted between August 2019 and January 2022 at Nigeria’s Jos University Teaching Hospital (JUTH). The study population consisted of women who were already enrolled in the United States National Cancer Institute U54CA221205 grant, titled “Epigenomic Biomarkers of HIV-Associated Cancers in Nigeria,” focusing on CC. Women were diagnosed with both low- and high-grade squamous intraepithelial lesions. In total, 101 women were enrolled in this pilot study in the colposcopy unit at JUTH in a consecutive order until all of the available enrollment slots were filled.

Data and sample collection

This study also employed a semi-structured questionnaire to gather information on household assets and douching behaviors. The project 2 database for the epigenomic biomarker of HIV-associated cancers in Nigeria was used to gather more information about the women’s backgrounds, number of children, age at first CC screening, and awareness of CC screening. By using such a broad data collection technique, we were able to identify a wide range of characteristics that could potentially influence CC outcomes. We also calculated the body mass index (BMI) of the study participants by measuring their height and weight. All information obtained was coded and kept confidential. We obtained from all enrolled participants a cervicovaginal swab for the assessment of HPV and STIs in a private room furnished with a gynecological examination bed at the colposcopy clinic. Participants were placed in the dorsal lithotomy posture for the swab collection. After a sterile speculum was inserted, a cytology brush was gently placed into the area of the cervix where changes occur and rotated to gather cervical cells. The samples were properly placed in a preservation medium before transportation. Before processing, the collected swabs were stored at -80°C.

Laboratory analysis

The University of Jos/JUTH Genomics and Postgraduate Research Laboratory, College of Health Sciences, was utilized for both HPV detection/genotyping and STI analyses. To ensure everything worked well before we extracted DNA, we added beta-mercaptoethanol to the genomic lysis buffer. The DNA was extracted from a 400 μL sample using the Quick-DNA™ Miniprep Kit (Zymo Research); the nucleic acids were collected in a final volume of 100 μL, and the purified DNA was stored at -80°C for further examination.

We used the HPV 28 Anyplex II Seegene real-time polymerase chain reaction (RT-PCR) to look for HPV DNA. This test can identify about 28 different types of HPV, 19 of which are high-risk and 9 are low-risk. We used two TOMs (TOCE Oligo Mixes), as directed by the manufacturer. The TOM A set contains primer DNA for 14 high-risk HPV types (16, 18, 31, 33, 35, 39, 45, 51, 52, 56, 58, 59, 66, 68), while the TOM B set includes primer DNA for five moderate-risk types (26, 53, 69, 73, 88) and nine low-risk HPV DNA (11, 34, 40, 42, 43, 44, 54, 61, 70). We used the Seegene Viewer software along with the CFX96 Real Time PCR (Bio-Rad) system to find and analyze the molecular data. The Seegene Viewer software and the compatible CFX96 Real Time PCR (Bio-Rad) system were utilized for detection, molecular data analysis, and interpretation.

To test for the presence of STIs, we used the Allplex STI Essential Assay. This is a real-time PCR assay that permits simultaneous amplification and detection of target nucleic acids of *C. trachomatis* (CT), *Neisseria gonorrhoeae* (NG), *Mycoplasma genitalium* (MG), *Mycoplasma hominis* (MH), *Ureaplasma urealyticum* (UU), *Ureaplasma parvum* (UP), *Trichomonas vaginalis* (TV), and internal control (IC). We conducted RT-PCR using a CFX-96 real-time thermocycler and followed the manufacturer’s guide for data analysis using the Seegene Viewer.

We also used the Seegene kit to perform multiplex RT-PCR (mPCR), which allowed us to amplify and detect the target nucleic acids of HSV-1 and 2, *Haemophilus ducreyi*, Cytomegalovirus, *Lymphogranuloma venereum*, *Treponema pallidum* (TP), varicella-zoster virus, and an IC. The Allplex Seegene kit uses special MuDT™ technology to give multiple threshold cycle (Ct) readings in one fluorescence channel without needing to analyze melt curves on RT-PCR machines. Following the manufacturer’s instructions, we performed the assay in a final reaction volume of 20 µL (15 µL master mix and 5 µL template), which included 5 µL of extracted template DNA. We viewed the CFX96TM test results in Seegene software.

Data management and analysis

Descriptive statistics were used to summarize the characteristics of study participants. Continuous variables were reported as median and interquartile range (IQR), and discrete variables as number and percentage. We examined the basic traits of study participants to determine if there was a link between having any HPV infection or STIs, using the chi-squared test or Fisher’s test for categorical variables and the Student’s t-test or Mann-Whitney U test for continuous variables. We used bivariate analysis to determine how ongoing hrHPV, STIs, and other factors are related by using the chi-square or Fisher’s exact test for categorical variables and the t-test for continuous variables. Associations producing a p-value <0.05 were considered statistically significant. A multivariate logistic regression model was used to determine STIs and other factors that were strongly linked to persistent hrHPV infection. All data were managed using the Research Electronic Data Capture (REDCap) database hosted at the University of Jos [13]. The primary dependent variables included the detection of cervical hrHPV and the presence of STIs. The independent variables comprised sociodemographic characteristics such as age, marital status, income, occupation, educational level, BMI, condom use, duration of HIV infection, reported lifetime number of sexual partners, and parity, obtained from the U54 database. Variables with a p-value <0.4 after univariate crude odds ratio (OR) and key epidemiological variables in previous studies were selected for multivariate analysis. Being a nested study, with data in RedCap, there were no cases of missing data. We used the variance inflation factor (VIF) to check for multicollinearity, and, in our study, the dummy- coded variables were not correlated. For statistical analysis, we captured 14 high-risk HPV and five moderate-risk HPV as hrHPV types. Data analysis was done using R and STATA version 17 (StataCorp., College Station, TX, USA).

Ethical considerations

Before we began collecting samples, we ensured that all participants had provided written informed consent for the study. The Health Research Ethical Review Committees of JUTH in Nigeria and Northwestern University in Chicago, Illinois, United States of America, approved the project (approval number: JUTH/DCS/IREC/127/XXX/2121).

## Results

In this study, we enrolled a total of 101 HIV-positive women with and without cervical precancer and normal Pap smear results. The median age from the summary statistics of the cohort data analysis was 50.0 years (IQR = 43-57), with a minimum and maximum age of 27 and 76 years, respectively. The participants’ characteristics and hrHPV status are shown in Table 1.

**Table 1:** Participants characteristics and high-risk HPV infection. hr-HPV = high-risk human papillomavirus; BMI = body mass index; HIV = human immunodeficiency virus

Exposure variables	Hr-HPV-negative, n (%) (n = 43)	Hr-HPV-positive, n (%) (n = 58)	Total (n = 101)	χ^2^	P-value
Age (years)				1.26	0.261
>45	25 (58.1)	40 (69.0)	65 (64.4)		
≤45	18 (41.9)	18 (31.0)	36 (35.6)		
Marital status				0.66	0.418
Married/Cohabiting	19 (44.2)	21 (36.2)	40 (39.6)		
Separated/Divorced/Widowed	24 (55.8)	37 (63.8)	61 (60.4)		
Occupation status				1.12	0.289
Employed	31 (72.1)	47 (81.0)	78 (77.2)		
Unemployed	12 (27.9)	11 (19.0)	23 (22.8)		
Education level				1.19	0.552
Primary or none	14 (32.6)	23 (39.7)	37 (36.6)		
At least secondary	17 (39.5)	17 (29.3)	34 (33.7)		
Post-secondary	12 (27.9)	18 (31.0)	30 (29.7)		
Income (USD)				0.03	0.852
>100	17 (39.5)	24 (41.4)	41 (40.6)		
≤100	26 (60.5)	34 (58.6)	60 (59.4)		
BMI				2.62	0.106
Normal	27 (62.8)	27 (46.6)	54 (53.5)		
Overweight	16 (37.2)	31 (53.4)	47 (46.5)		
Parity				4.59	0.101
≤1	9 (20.9)	10 (17.2)	19 (18.8)		
2-4	14 (32.6)	31 (53.5)	45 (44.6)		
>4	20 (46.5)	17 (29.3)	37 (36.6)		
Total number of lifetime sexual partners				0.48	0.491
<3	23 (53.5)	27 (46.6)	50 (49.5)		
≥3	20 (46.5)	31 (53.4)	51 (50.5)		
Consistency of condom use				0.74	0.692
Always	5 (11.6)	9 (15.5)	14 (13.9)		
Occasionally	10 (23.3)	10 (17.2)	20 (66.3)		
Never	28 (65.1)	39 (67.2)	67 (19.8)		
Age at first cervical screening (years)				1.01	0.314
<45	28 (65.1)	32 (55.2)	43 (42.6)		
≥45	15 (34.9)	26 (44.8)	58 (57.4)		
Duration of HIV infection (years)				1.62	0.203
>10	22 (51.2)	37 (63.8)	59 (58.4)		
≤10	21 (48.8)	21 (36.2)	42 (51.6)		

Of the participants, 40/101 (40%) had coinfections with hrHPV and other STIs, whereas 61/101 (60%) did not. Those who had precancer lesions had the highest hrHPV/STI co-infection of 27/40 (68%) (Table 2).

**Table 2:** High-risk HPV association with sexually transmitted infections among women living with HIV. hr-HPV = high-risk human papillomavirus; HIV = human immunodeficiency virus; STI = sexually transmitted infection

Exposure indices	hr-HPV-negative, n (%)	hr-HPV-positive, n (%)	Total (n = 101)	χ^2^	P-value
Any STI	18 (31.0)	40 (69.0)	58 (57.4)	7.42	0.006
Non-ulcerative STIs	15 (34.9)	36 (62.1)	51 (50.5)	7.30	0.007
*Neisseria gonorrhoeae*+	0	1 (1.7)	1 (1.0)	0.75	0.387
*Mycoplasma genitalium* +	2 (4.7)	4 (6.9)	6 (5.9)	0.22	0.637
*Trichomonas vaginalis*+	3 (7.0)	9 (15.5)	12 (11.9)	1.72	0.190
*Ureaplasma parvum* +	9 (20.9)	22 (37.9)	31 (30.7)	3.36	0.067
*Ureaplasma urealyticum*+	5 (11.6)	15 (25.9)	20 (19.8)	3.15	0.076
Multiple non-ulcerative STIs				8.07	0.018
Absent	28 (65.1)	22 (37.9)	50 (49.5)		
Single	12 (27.9)	24 (41.1)	36 (35.6)		
Multiple	3 (7.0)	12 (20.7)	15 (14.9)		
Any ulcerative STIs	15 (34.9)	38 (65.5)	53 (52.5)	9.29	0.002
Cytomegalovirus +	3 (7.0)	5 (8.6)	8 (7.9)	0.09	0.762
*Haemophilus ducreyi* +	5 (11.6)	14 (24.1)	19 (18.8)	2.53	0.112
Herpes simplex virus type 1 +	4 (9.3)	14 (24.1)	18 (17.8)	3.71	0.054
Herpes simplex virus type 2+	0	1 (1.7)	1 (1.0)	0.75	0.387
*Lymphogranuloma venereum* +	6 (14.0)	22 (37.9)	28 (27.7)	7.09	0.008
Varicella-zoster virus +	8 (18.6)	19 (32.8)	27 (26.7)	2.53	0.112
Multiple ulcerative STIs				7.18	0.028
Absent	28 (65.1)	20 (34.5)	48 (47.5)		
Single	7 (16.3)	13 (22.4)	20 (19.8)		
Multiple	8 (18.6)	25 (43.1)	33 (32.7)		

The presence of any STI was detected in 40/58 (69%) women who tested positive for hrHPV (p = 0.006). Among the women who were hrHPV-positive, 36/58 (62%) had non-ulcerative STIs, and 38/58 (65%) had ulcer-causing STIs. The association between hrHPV and any STI, non-ulcerative STIs, and genital ulcer-causing STIs was statistically significant (p < 0.05). Multiple STIs were more common among hrHPV-positive women than hrHPV-negative women. In univariate analyses of any ulcerative and non-ulcerative STIs, *M. genitalium*, HSV 1, and *L. venereum* were associated with hrHPV infection (Table 4). In multivariable models adjusting for demographics, the OR (95% CI; p-value) for any ulcerative STI was 10 (3.7-38.9, p < 0.005). Other significant associations in the univariable models became insignificant in the multivariable model (Table 3).

**Table 3:** High-risk HPV univariate and multivariate logistic analysis. HPV = human papillomavirus; HIV = human immunodeficiency virus; STI = sexually transmitted infection; aOR = adjusted odds ratio; CI = confidence interval

	Univariate analysis			Multivariate analysis		
Risk factors	OR	95% CI	P-value	aOR	95% CI	P-value
Age (years)						
>45	1					
≤45	1.68	0.74–3.85	0.218	3.62	1.05–13.72	0.047
Marital status						
Married/Cohabiting	1					
Separated/Divorced/Widowed	1.22	0.54–2.72	0.630	-	-	-
Occupation status						
Employed	1					
Unemployed	0.54	0.21–1.36	0.192	-	-	-
Education level						
Primary or none	1					
At least Secondary	0.76	0.30–1.94	0.569	-	-	-
Post-secondary	1.14	0.43–3.07	0.789	-	-	-
Income (USD)						
>100	1					
≤100	0.81	0.36–1.80	0.606	-	-	-
Parity						
≤1	1					
2-4	1.80	0.60–5.43	0.292	1.10	0.29–4.08	0.884
>4	0.69	0.22–2.09	0.506	0.05	0.01–0.33	0.003
Age at sexual debut (years)						
≥18	1					
<18	1.23	0.56–2.78	0.606	-	-	-
HIV duration						
≥10	1					
<10	0.99	0.91–1.08	0.843	0.91	0.80–1.02	0.107
Non-ulcerative STIs	3.02	1.35–6.96	0.008	-	-	-
Mycoplasma genitalium	1.65	0.31–12.34	0.572	-	-	-
Trichomonas vaginalis	2.68	0.74–12.68	0.159	-	-	-
Ureaplasma parvum	2.10	0.88–5.26	0.101	-	-	-
Ureaplasma urealyticum	2.93	1.03–9.69	0.056	-	-	-
Multiple non-ulcerative STIs						
Absent	1					
Single	5.52	1.53–26.51	0.016	-	-	-
Multiple	2.44	1.02–6.02	0.047	-	-	-
Genital ulcerative STIs	3.53	1.57–8.21	0.003	10.43	3.37–38.94	0.000
Cytomegalovirus	1.37	0.32–7.00	0.677	-	-	-
Haemophilus ducreyi	1.97	0.70–6.06	0.212	-	-	-
Herpes simplex virus type 1	3.42	1.12–12.84	0.043	-	-	-
Lymphogranuloma venereum	3.26	1.28–9.13	0.017	-	-	-
Varicella-zoster virus	1.89	0.77–4.93	0.174	-	-	-
Multiple genital non-ulcerative STIs						
Absent	1					
Single	4.07	1.60–11.06	0.004	-	-	-
Multiple	2.83	0.98–8.79	0.060	-	-	-

**Figure 1:**
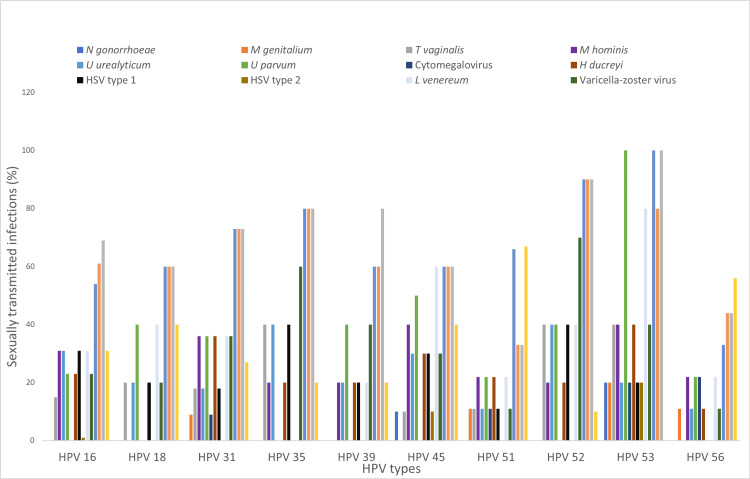
Prevalence of sexually transmitted infections coinfection with HPV types. HPV = human papillomavirus

Figure 1 shows hrHPV/STI coinfection with specific STI types. All participants who tested positive for *N. gonorrhea* and HSV 2 also had at least one hrHPV infection, followed by 79% for *L. venereum,* 78% for HSV 1, and 75% for both *T. vaginalis* and *U. urealyticum*.

**Figure 2:**
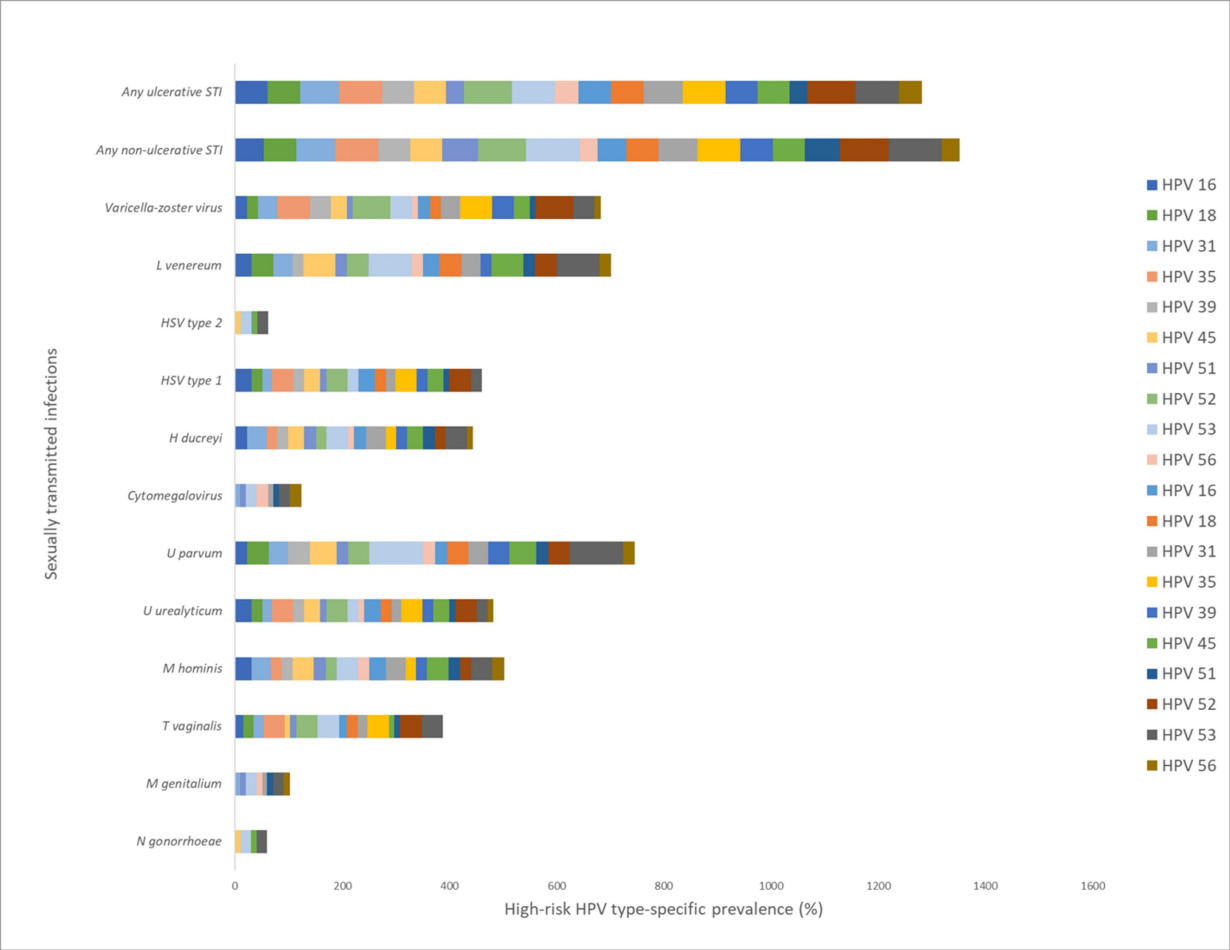
Relationship between hrHPV specific types and individual STIs among women living with HIV in Jos, Nigeria hrHPV = high-risk human papillomavirus; STI = sexually transmitted infection; HIV = human immunodeficiency virus; HSV = herpes simplex virus

Figure 2 illustrates the relationship between hrHPV-specific types and individual STIs. Overall, 80% (4/5) of patients with a positive test for *L. venereum* had hrHPV type 53 (p = 0.007). HPV 53 was also associated with HSV 2 (p = 0.006), *U. parvum* (p = 0.001), *T. vaginalis* (p = 0.046), and *N. gonorrhea* (p = 0.000).

## Discussion

This study examines the relationship between hrHPV infections and other STIs among WLWH in Jos, Nigeria. Women with HIV face an elevated risk of contracting HPV and STIs, which subsequently can increase the likelihood of developing HPV-related cancers in susceptible anatomical sites [14,15]. The 40% prevalence of hrHPV-STI among WLWH in this study supports earlier research, indicating that STIs, including *Chlamydia trachomatis* and HSV 2, may act as cofactors in the acquisition of HPV and possibly in the persistence of the infection. The inflammation resulting from these infections may lead to cellular alterations that promote HPV persistence and increase the risk of cervical precancerous lesions and cancer [15-17].

Prior research indicates that women over 30 years old with hrHPV infection are at an increased risk of having underlying cervical pathology [18-20]. The participants in our study were between the ages of 27 and 76 years, which is in line with research showing that HPV-related cervical lesions can affect both reproductive-age and older individuals with HIV. This observation shows how important it is to have targeted interventions and awareness campaigns for this group of individuals.

Our analysis revealed that none of the sociodemographic variables exhibited a significant association with hrHPV infections in the study sample (p > 0.05). Additionally, parity, total lifetime sexual partners, age at first cervical screening, duration of HIV infection, and consistency of condom use also showed no statistical association with hrHPV infections. The results correspond with a study from Plateau State, except for primary education level, age at first cervical screening, and BMI [20]. However, several studies have reported results that differ from our findings. The discrepancies highlight the complexity of hrHPV infection dynamics and suggest that various sociodemographic and behavioral factors may differentially affect infection rates among distinct populations. Future research should aim to examine these factors in greater depth to identify specific prevention strategies. Lifetime number of sexual partners, consistent condom use, and duration of HIV infection are established risk factors for HPV infections globally [21]. Certain participants may have experienced reporting or recall bias.

This study identified no correlation between the duration of HIV infection among participants and hrHPV infection. Other studies indicate that HIV-positive women have a higher likelihood of acquiring and retaining HPV compared to their HIV-negative counterparts. The presence of HIV is associated with a threefold increase in the risk of HPV acquisition and a significantly lower rate of infection clearance [14,15,22]. The immunocompromised condition linked to HIV infection increases women’s susceptibility to hrHPV and diminishes their capacity to eliminate existing infections, consequently elevating their risk for persistence and CC. This underscores the essential role of regular screening and preventive strategies for women with HIV.

Associations were identified between hrHPV types and both ulcerative and non-ulcerative STIs. This finding aligns with earlier studies that identified multiple STIs as potential facilitators of HPV-related carcinogenesis, likely due to shared risk factors and transmission routes [5,6]. Coinfections may increase the risk of CC and facilitate the progression of cervical dysplasia in individuals positive for HPV [11]. The disruption of the cervical epithelium and alteration of the vaginal microbiome by other STIs can foster an environment conducive to the persistence of HPV infection. This disturbance may increase viral load and shedding, contributing to the persistence of HPV and potentially resulting in more severe health outcomes [23]. Comprehending these interactions is crucial for developing effective prevention strategies and treatment options. Simultaneous management of HPV and coinfections by healthcare providers can enhance patient outcomes and decrease the overall incidence of CC.

Certain HPV types were linked to multiple STIs, such as *L. venereum*, HSV 2, *T. vaginalis*, and *N. gonorrhoeae*. One significant finding indicates that 80% of patients with a positive *L. venereum* test were identified as having hrHPV type 53 (p = 0.007), suggesting a correlation between specific HPV types and certain STIs. Certain STIs are treatable, and effective treatment may substantially decrease the population-level risk of transmission, HPV persistence, and oncogenesis. Understanding the interactions between these infections can guide public health strategies aimed at reducing the incidence of STIs and HPV-related cancers.

In a multivariate analysis of risk factors, findings demonstrate that WLWH aged over 45 years exhibited an almost fourfold increase in the likelihood of hrHPV infection (adjusted odds ratio (aOR) = 3.62, p = 0.047). The heightened risk correlated with age corresponds with findings from various studies that emphasize the vulnerability of older women to enduring hrHPV infections. A systematic review indicated that the pooled prevalence of hrHPV among HIV-positive women in Nigeria is 71%, particularly within reproductive age groups and extending to 65 years of age [24]. Individuals under 30 years of age are at a higher risk of acquiring HPV due to earlier initiation of sexual activity and a higher number of sexual partners [23,24]. The identified factors underscore the necessity for focused screening and vaccination initiatives to reduce the effects of hrHPV across various age groups.

Our result indicates a notable association between parity and hrHPV infection. Women with more than four children exhibited a significantly lower incidence of hrHPV infection (aOR = 0.05, p = 0.003). This finding may suggest intricate biological mechanisms through which multiparous women undergo recurrent exposure to HPV, resulting in the formation of adaptive immune responses that facilitate viral control, or they may exhibit unique health-seeking behaviors, such as consistent cancer screenings, unlike nulliparous women [25]. These findings indicate that reproductive history may significantly influence women’s susceptibility to hrHPV, underscoring the necessity of incorporating parity into public health strategies designed to mitigate CC risks. Additional investigation is required to examine the biological and behavioral factors that underlie these observed differences.

Genital ulcerative STIs demonstrated a significant association with hrHPV infection, as evidenced by univariate (OR = 3.53, p = 0.003) and multivariate analyses (aOR = 10.43, p < 0.001). This aligns with prior studies demonstrating a synergistic relationship between hrHPV and other STIs, particularly genital ulcerative diseases such as HSV and syphilis [26,27]. The study found significant associations with multiple non-ulcerative STIs (p < 0.05), reinforcing the hypothesis that coinfections may enhance susceptibility to HPV. Further investigation is required to elucidate the underlying mechanisms that govern these associations. Examining the impact of coinfections on the progression and persistence of hrHPV may enhance the development of targeted prevention and treatment strategies.

The study revealed no significant correlation between marital status, profession, education level, and the incidence of hrHPV infection. Conversely, numerous studies conducted in Nigeria and elsewhere contest this lack of association [18,19,28], while others corroborate it [20,29]. The study also found no statistically significant link between how long someone has had HIV, how old they were when they first had sex, and the rate of hrHPV infection. This outcome is in line with earlier research [30]. Alternative studies indicate a correlation between these variables and hrHPV infection [23,24]. The conflicting findings draw attention to the complexity of the relationship between HIV and hrHPV infections, indicating a need for further investigation to elucidate these associations.

Our study highlights the importance of regular screening and monitoring for STIs in women living with HIV, as managing these infections effectively may reduce the incidence of hrHPV-related complications. Public health efforts to raise awareness and make preventive measures more accessible are important for lowering these risks. Public health initiatives should emphasize education regarding safe sexual practices and the advantages of vaccination to mitigate the overall incidence of HPV-related diseases. Future research should look at a wider range of factors, such as social and economic influences, to better understand the underlying dynamics of these infections.

Limitations

The small sample size of this study may limit the generalizability of the findings. Future research should increase the participant pool to provide a more comprehensive understanding of the dynamics of hrHPV coinfection with other STIs and associated risk factors. This study is also limited by its cross-sectional design, which restricts causal inference between HPV infection and STI coinfection because both exposure and outcome were measured simultaneously, preventing the assessment of HPV persistence; this persistence requires longitudinal follow-up to distinguish transient infections from persistent ones. Therefore, the findings should be interpreted as associations rather than evidence of cause-and-effect relationships. Additionally, the participants in our study were exclusively HIV-positive women, which may further limit the generalizability of the findings to wider populations. To improve the validity of future research, a larger and more diversified sample could enhance the comprehension of the epidemiology and risk factors of hrHPV and STI coinfection.

## Conclusions

The results of this study show that HPV coinfection with other STIs is common. Ongoing efforts through screening for these STIs are necessary to address curable STIs in WLWH to mitigate hrHPV infection and its associated risks of persistence and progression to malignancy. Addressing these coinfections improves health outcomes for WLWH and supports public health initiatives focused on reducing the overall burden of HPV-related diseases. Collaborative strategies encompassing education, screening, and vaccination are crucial for achieving sustained success in the fight against these infections.
